# Supplementary material for: Novel Driver Strength Index highlights important cancer genes in TCGA PanCanAtlas patients
Source: PeerJ. 2022 Aug 11;10:e13860. doi: 10.7717/peerj.13860 (PMC9375969; doi:10.7717/peerj.13860)
Supplement: Supplemental Information 2 [file peerj-10-13860-s002.zip › COHORTS/genes plots/detailed/2021_8_16_14_9_distribution_events_detailed_females_16.pdf]

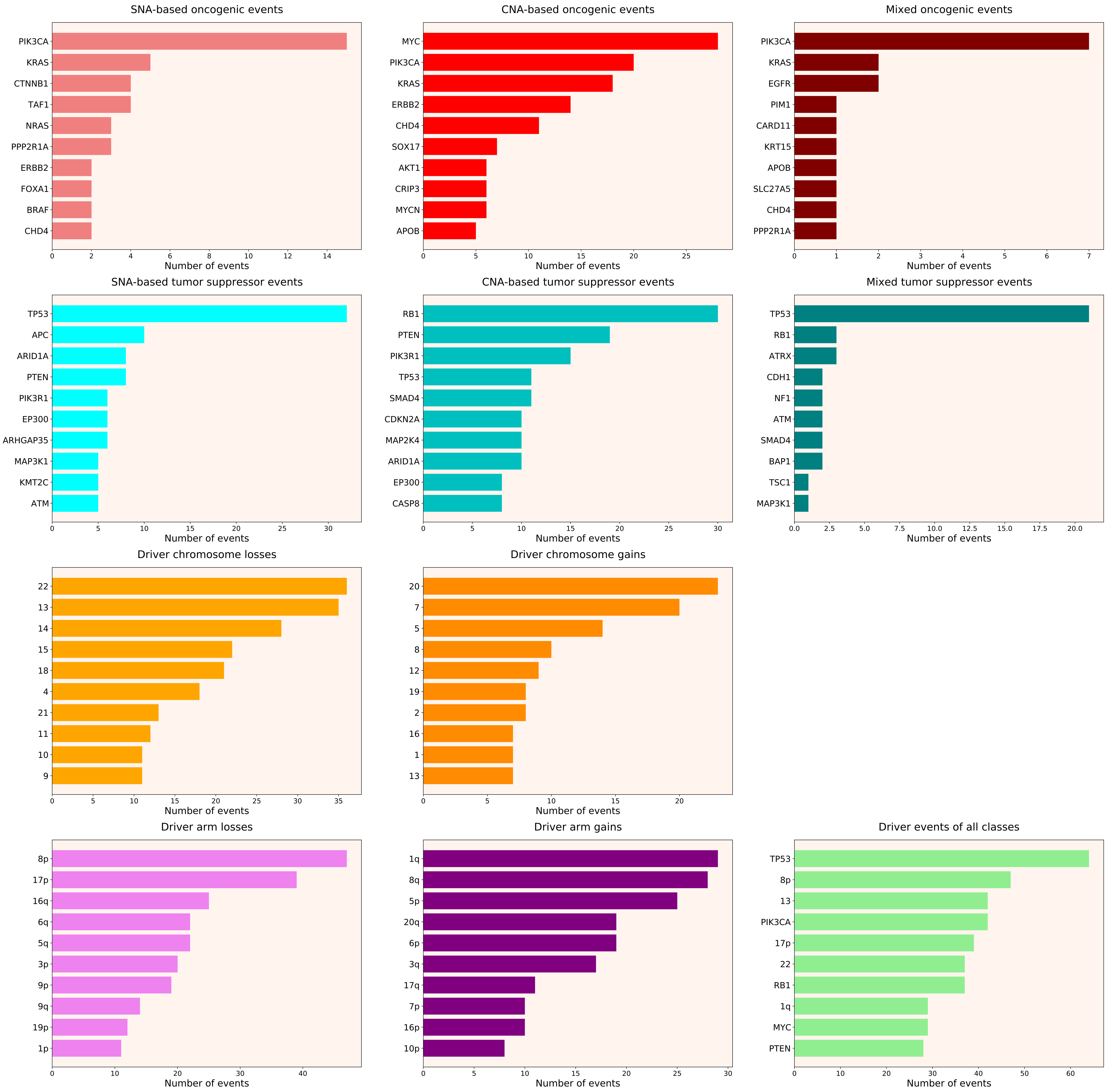

SNA-based oncogenic events
PIK3CA
KRAS
CTNNB1
TAF1
NRAS
PPP2R1A
ERBB2
FOXA1
BRAF
CHD4
0 2 4 6 8 10 12 14
Number of events
CNA-based oncogenic events
MYC
PIK3CA
KRAS
ERBB2
CHD4
SOX17
AKT1
CRIP3
MYCN
APOB
0 5 10 15 20 25
Number of events
Mixed oncogenic events
PIK3CA
KRAS
EGFR
PIM1
CARD11
KRT15
APOB
SLC27A5
CHD4
PPP2R1A
0 1 2 3 4 5 6 7
Number of events
SNA-based tumor suppressor events
TP53
APC
ARID1A
PTEN
PIK3R1
EP300
ARHGAP35
MAP3K1
KMT2C
ATM
0 5 10 15 20 25 30
Number of events
CNA-based tumor suppressor events
RB1
PTEN
PIK3R1
TP53
SMAD4
CDKN2A
MAP2K4
ARID1A
EP300
CASP8
0 5 10 15 20 25 30
Number of events
Mixed tumor suppressor events
TP53
RB1
ATRX
CDH1
NF1
ATM
SMAD4
BAP1
TSC1
MAP3K1
0.0 2.5 5.0 7.5 10.0 12.5 15.0 17.5 20.0
Number of events
Driver chromosome losses
22
13
14
15
18
4
21
11
10
9
0 5 10 15 20 25 30 35
Number of events
Driver chromosome gains
20
7
5
8
12
19
2
16
1
13
0 5 10 15 20
Number of events
Driver arm losses
8p
17p
16q
6q
5q
3p
9p
9q
19p
1p
0 10 20 30 40
Number of events
Driver arm gains
1q
8q
5p
20q
6p
3q
17q
7p
16p
10p
0 5 10 15 20 25 30
Number of events
Driver events of all classes
TP53
8p
13
PIK3CA
17p
22
RB1
1q
MYC
PTEN
0 10 20 30 40 50 60
Number of events
